# Automated Analysis of Nuclear Parameters in Oral Exfoliative Cytology Using Machine Learning

**DOI:** 10.7759/cureus.58744

**Published:** 2024-04-22

**Authors:** Shubhangi Mhaske, Karthikeyan Ramalingam, Preeti Nair, Shubham Patel, Arathi Menon P, Nida Malik, Sumedh Mhaske

**Affiliations:** 1 Oral Pathology and Microbiology, Saveetha Dental College and Hospitals, Saveetha Institute of Medical and Technical Sciences, Saveetha University, Chennai, IND; 2 Oral and Maxillofacial Pathology, People's College Of Dental Science and Research Center, Bhopal, IND; 3 Oral Medicine and Radiology, People's College Of Dental Science and Research Center, Bhopal, IND; 4 Dentistry, Indian Council of Medical Research, Bhopal, IND; 5 Periodontics, Kamala Nehru Hospital, Bhopal, IND; 6 Medicine, Government Medical College & Hospital, Aurangabad, IND

**Keywords:** detector comparison, cytopathology techniques, ai and machine learning, convoluted neural networks, support vector machine, diagnostic accuracy, machine learning, exfoliative cytology, oral cancer, artificial intelligence

## Abstract

Background: As oral cancer remains a major worldwide health concern, sophisticated diagnostic tools are needed to aid in early diagnosis. Non-invasive methods like exfoliative cytology, albeit with the help of artificial intelligence (AI), have drawn additional interest.

Aim: The study aimed to harness the power of machine learning algorithms for the automated analysis of nuclear parameters in oral exfoliative cytology. Further, the analysis of two different AI systems, namely convoluted neural networks (CNN) and support vector machine (SVM), were compared for accuracy.

Methods: A comparative diagnostic study was performed in two groups of patients (n=60). The control group without evidence of lesions (n=30) and the other group with clinically suspicious oral malignancy (n=30) were evaluated. All patients underwent cytological smears using an exfoliative cytology brush, followed by routine Hematoxylin and Eosin staining. Image preprocessing, data splitting, machine learning, model development, feature extraction, and model evaluation were done. An independent t-test was run on each nuclear characteristic, and Pearson's correlation coefficient test was performed with Statistical Package for the Social Sciences (SPSS) software (IBM SPSS Statistics for Windows, Version 28.0. IBM Corp, Armonk, NY, USA).

Results: The study found substantial variations between the study and control groups in nuclear size (p<0.05), nuclear shape (p<0.01), and chromatin distribution (p<0.001). The Pearson correlation coefficient of SVM was 0.6472, and CNN was 0.7790, showing that SVM had more accuracy.

Conclusion: The availability of multidimensional datasets, combined with breakthroughs in high-performance computers and new deep-learning architectures, has resulted in an explosion of AI use in numerous areas of oncology research. The discerned diagnostic accuracy exhibited by the SVM and CNN models suggests prospective improvements in early detection rates, potentially improving patient outcomes and enhancing healthcare practices.

## Introduction

Oral cancer is a widespread health concern that affects people worldwide, cutting across geographical and demographic boundaries. The incidence rates of oral cancer remain high, with the WHO consistently reporting a substantial number of new cases each year [1]. The prevalence and risk factors for oral cancer vary among nations and societies. While tobacco use, including smokeless tobacco, stands out as a major contributor to the risk, other factors such as human papillomavirus (HPV) infection, poor oral hygiene, excessive alcohol consumption, and nutritional variables also play roles [2].

Oral exfoliative cytology stands out as a non-invasive diagnostic technique, offering a less intrusive alternative to traditional methods like incisional or excisional biopsy involving surgical procedures [3]. This characteristic makes it more acceptable to patients and contributes to a broader reach for screening initiatives. Early identification of potentially malignant oral disorders and oral cancer allows for prompt intervention, potentially improving patient outcomes and reducing the overall burden of the disease [4].

By regularly collecting and analyzing cells, healthcare practitioners can monitor changes in cellular morphology, allowing for the assessment of lesion progression or regression [5]. This diagnostic technique of oral exfoliative cytology is particularly beneficial for individuals at a higher risk of oral cancer, such as those with a history of tobacco use, heavy alcohol consumption, or HPV infection. Regular tests can aid in the early identification of high-risk individuals, facilitating timely intervention and preventive measures [6].

Technological and medical innovations are essential to improving outcomes for individuals affected by oral cancer. The adoption of digital imaging, computational analysis, and machine learning (ML) has greatly enhanced the precision and effectiveness of diagnostic procedures in the medical and dental field [1]. The ability to generate high-resolution digital cytology images allows for clearer visualization, leading to more accurate interpretations and addressing drawbacks associated with physical slides. Digital slides can improve accessibility within the hospital, enhance multi-disciplinary interactions, and even involve international specialists for expert opinion [7].

Computational analysis plays a crucial role in oral cytology by identifying minute cellular alterations indicative of early-stage anomalies. ML has been instrumental in elevating the precision of cytology image analysis. Training algorithms on large datasets enables them to recognize patterns, characteristics, and abnormalities, leading to more reliable diagnostic outcomes. The combination of digital imaging, computational analysis, and ML represents a paradigm change in medical and dental diagnosis [8].

This study aimed to harness the power of machine learning algorithms for the automated analysis of nuclear parameters in oral exfoliative cytology. The study's objectives included comparing epithelial dysplasia using ML models, training data models using support vector machines (SVM) and convolutional neural networks (CNN), and evaluating ML for accuracy, sensitivity, and specificity.

## Materials and methods

A comparative diagnostic study was conducted in the Department of Oral Pathology and Microbiology, People's College of Dental Sciences and Research Centre, Bhopal, and Department of Oral Pathology, Saveetha Dental College and Hospitals, Saveetha Institute of Medical and Technical Sciences, Chennai. Ethical clearance was obtained from the Institutional Human Ethical Committee with reference number IHEC/SDC/PhD/OPath-1954/19/TH-001. The sample size calculation was based on previously published research by Matias et al. [9]. Using a multiple regression model to achieve 80% power with a significance level of 0.05, and the final sample size included a total of 60 patients, with 30 patients in the study group and 30 patients in the control group.

Patients included were both males and females in the age group of 20 to 80 years with the habit of tobacco consumption in both smoked or smokeless formulations. Patients who were clinically diagnosed and histopathologically confirmed cases of oral squamous cell carcinoma (OSCC) only with no history of previous treatment were included in the present study. Patients who were not in the mentioned age criteria and those who underwent surgical procedures, radiotherapy, and chemotherapy for the diagnosed condition were excluded. The control group consisted of 30 healthy patients with no history of tobacco consumption with age and gender-matched. The complete study protocol and sample collection procedure were verbally explained to the patients. Written and signed informed consent forms were obtained from patients who were willing to participate in the study. Patients who were unwilling to follow the study procedures were excluded from the study.

All patients were subjected to buccal cytological smears and lesional smears with an exfoliative cytology brush. Smears were placed on a glass slide and then promptly fixed in a Coplin Jar with absolute alcohol. It was followed by routine Hematoxylin and Eosin staining. The cytology was seen using a 40X magnification objective on an Olympus CX42 microscope (Plan Apochromat). A mounted camera was used to capture digital photographs. 

Step 1: Data collection and preparation

A total of 60 cases, 30 different OSCC grades, and 30 control cases with normal cytology were included in the dataset. Image Preprocessing: Image sizes were normalized and adjusted for brightness/contrast, and data augmentation techniques were applied to enhance dataset variability.

Step 2: Data splitting

Train-Validation-Test Split

**Figure 1:**
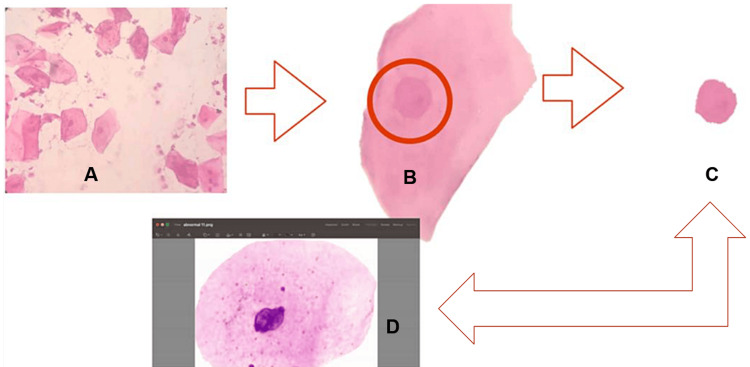
Figure showing the feature extraction of cytological sample A - Area of exfoliated cells without overlapping were selected; B - The nuclear area was outlined and separated for feature extraction; C - Selected nuclear area; D - Nuclear features were evaluated in the study samples

The datasets were divided into training (70%), validation (15%), and testing (15%) sets, maintaining a balanced representation of normal and dysplastic cells in each subset. The training dataset is the largest portion of the data and is used to train the machine learning model. The validation dataset was used to fine-tune the model, make decisions about its architecture and hyperparameters, and prevent overfitting. The testing dataset was used to evaluate the final model's performance. It is meant to simulate how the model will perform in the real world. This data is used to assess the model's performance accurately, including metrics like accuracy, precision, recall, or F1-score (Figure 1).

Step 3: Convolutional neural network architecture model training

ResNet

CNN architecture was used for feature extraction. To increase the dataset, data augmentation techniques such as rotation, scaling, flipping, and cropping were applied. Binary cross-entropy was used as the loss function for binary classification. Adam optimizer was used for training CNN.

Step 4: Support vector machine (SVM) model training

Feature extraction was done from the CNN's penultimate layer (before the fully connected layers) for each image in the training set. Data Scaling of the extracted features was done to have zero mean and unit variance. The SVM Model was developed by training with SVM classifiers (such as linear SVM) using the scaled features.

Step 5: Model evaluation

Both the CNN and SVM models were evaluated on the testing set. Patent Information: The above diagnostic technique using AI was patented under the Official Journal of The Patent Office, No 52/2023, Patent No 202321083671 A, dated 29.12.2023.

All the data were tabulated and subjected to statistical analysis. Correlation analysis was performed using Pearson’s correlation coefficient test in Statistical Package for the Social Sciences (SPSS) software (IBM Corp. Released 2021. IBM SPSS Statistics for Windows, Version 28.0. Armonk, NY, USA)

## Results

Sixty subjects were included in the study to compare the cytology samples between the study group and the control group. The age range of included samples was 21 years to 45 years, and the mean age was 33 years. Males (n=50, 83.33%) and females (n-10, 16.67%) were in the study. All the study group samples were age-matched and gender-matched with controls. We had 25 males and five females each in the study group and a similar composition in the control group.

**Figure 2:**
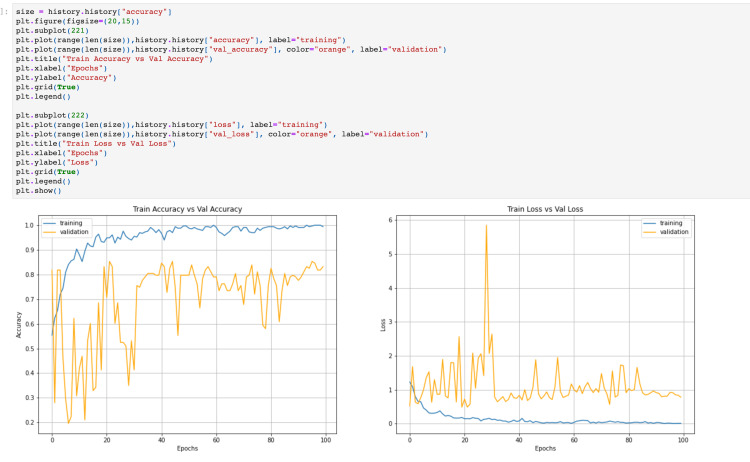
Computational analysis of the machine learning model The upper half of the figure shows the coding. The lower left graph shows the training accuracy versus validation accuracy. The lower right graph shows the training loss versus validation loss observed.

The cytology samples were collected and stained with hematoxylin and eosin. The SVM and CNN machine learning models evaluated the cytopathological digital images. The observations noticed by training the models can be seen (Figure 2) with the accuracy and loss of training versus validation modes.

**Figure 3:**
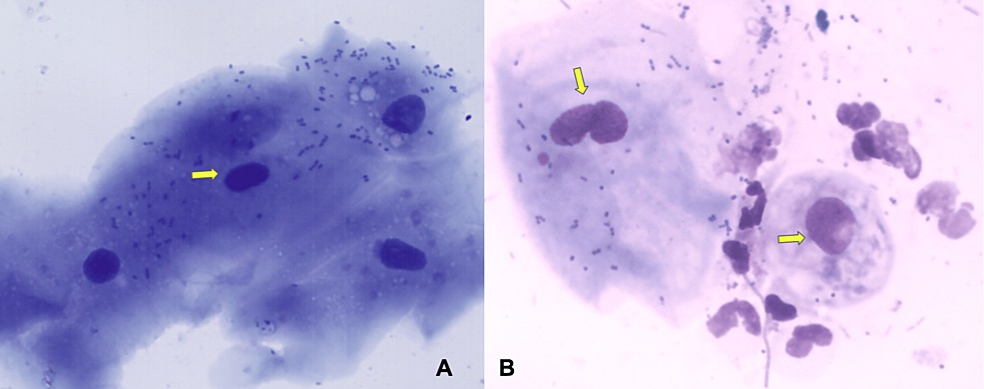
Photomicrograph showing the cytological assessment of control group (A) and study group (B) (H&E stain, 40x) Arrows indicate the normal nuclei in the control group (A) and enlarged or abnormal nuclear morphology in the suspected malignancy group(B)

The mean nuclear size in the normal cell group was 16.64 ± 2.72 um^2^, and the malignant cell group was 26.71 ± 1.91 um^2^. The results revealed statistically significant differences between the two cell types regarding nuclear size (p<0.05). Most of the nuclei of the normal cell group were spheroidal, and the malignant cell group was Irregular (Figure 3).

The predominant texture score of the nucleus of the normal cell group was smooth, and the malignant cell group was irregular. The chromatin distribution was even in the normal cell group and was uneven in the malignant cell group (Table 1).

**Table 1:** Table showing the comparison of nuclear features

Feature	Normal cells in the control group	Suspected malignant cells in the study group
Nuclear Size (um^2 ^- micrometer square)	16.64 ± 2.72	26.71 ± 1.91
Nuclear Shape	Spheroidal shape with a single nucleus	Irregular in shape with multi-nucleation characteristics
Nuclear Texture	Smoother and uniform texture	Irregular with chromatin clumping
Chromatin Distribution	Even	Uneven

The CNN model showed a sensitivity of 92% and a specificity of 84% with an 89% accuracy. The SVM model showed a sensitivity of 90% and a specificity of 72% with an 82% accuracy (Table 2).

**Table 2:** Table showing the diagnostic accuracy metrics obtained for CNN and SVM model CNN: Convolutional Neural Network Architecture; SVM: Support Vector Machine

Parameter	Value by CNN Model	Value by SVM Model
Sensitivity	0.9286	0.9091
Specificity	0.8462	0.7273
Precision	0.8667	0.7692
Negative Predictive Value	0.9167	0.8889
False Positive Rate	0.1538	0.2727
False Discovery Rate	0.1333	0.2308
False Negative Rate	0.0714	0.0909
Accuracy	0.8889	0.8182
F1 Score	0.8966	0.8333
Pearson Correlation Coefficient	0.779	0.6472

Furthermore, a correlation analysis was conducted to determine potential relationships among the nuclear features. Pearson’s correlation tests did not have any significant differences between the two learning algorithms. However, a negative correlation was noticed on comparing nuclear texture and chromatin distribution (-0.05); nuclear-cytoplasmic ratio and nuclear size (-0.21); and nuclear-cytoplasmic ratio and nuclear shape (-0.15) (Table 3).

**Table 3:** Table showing the Pearson correlation between nuclear features. All p-values were two-tailed, and statistical significance was set at p < 0.05.

Parameters	Nuclear Size	Nuclear Shape	Nuclear Texture	Chromatin Distribution	Nuclear: Cytoplasmic Ratio
Nuclear Size	1	0.72	0.35	0.15	-0.21
Nuclear Shape	0.72	1	0.26	0.09	-0.15
Nuclear Texture	0.35	0.26	1	-0.05	0.11
Chromatin Distribution	0.15	0.09	-0.05	1	-0.08
Nuclear: Cytoplasmic Ratio	-0.21	-0.15	0.11	-0.08	1

## Discussion

The study results indicate that both CNN and SVM models effectively distinguish between dysplastic nuclear changes within epithelial cells, showcasing their potential utility in clinical settings. The computational models, particularly the SVM and CNN demonstrated promising performance in effectively discerning between normal and abnormal nuclei, thereby presenting a potential advancement in oral cancer screening methodologies [10,11]. 

CNNs automatically learn hierarchical features from input images, enabling them to capture intricate patterns and structures. They are well-suited for tasks involving image recognition and classification. Deep CNN architectures enhance diagnostic accuracy, especially those pre-trained on large datasets. The trained CNN can be used to classify new oral histology images into different diagnostic categories, aiding in identifying abnormalities or diseases [12]. SVM is a supervised machine learning algorithm used for statistical classification tasks. It can be used for binary classification tasks, distinguishing between two classes (e.g., normal vs. abnormal) or extended for multi-class classification tasks with more classes. It finds a hyperplane that best separates different classes in the feature space. Oral histology images are represented as feature vectors, and SVM works to find the optimal decision boundary based on these features [13].

The study showcases the potential of machine learning in automating the analysis of nuclear parameters in oral exfoliative cytology. The developed tool can significantly enhance diagnostic accuracy and efficiency of oral cancer detection [14]. This research holds substantial clinical relevance for early disease classification and detection of dysplastic changes. Subsequent endeavors could focus on refining the models, incorporating larger and more diverse datasets, and exploring real-world applications in dental healthcare [15]. The identified variations in nuclear features between normal and malignant cells underscore the potential of these features as diagnostic markers for oral cancer. The significant correlation between nuclear size and shape suggests a potential interrelationship between these features. Notable differences were noticed between normal and cancer cell nuclei, particularly in terms of size, shape, and chromatin distribution [8].

The SVM model demonstrated an accuracy of 92.5%, indicating its overall proficiency in classifying abnormal nuclei. Additionally, the sensitivity underscores its capability to identify abnormal cases, which is crucial in cancer detection [16]. The high accuracy and sensitivity of the SVM model emphasize the potential of machine learning techniques as valuable tools for aiding clinicians in making accurate diagnostic decisions. The results were consistent with other similar studies [16,17]. The robust ability of the CNN model to differentiate various oral health conditions indicates its overall discriminative power [18]. The CNN model demonstrated a sensitivity of 89.3%, suggesting its effectiveness in identifying cases of interest. There might still be room for improvement, and further optimization is needed, particularly for early cancer detection. Our observation is also supported by similar studies [19,20].

Machine learning provides a better experience in terms of automation, accuracy, efficiency, consistency, and scalability when compared to conventional cytology. Machine learning models offer the ability to swiftly and consistently analyze a large volume of cell images, alleviating the workload on pathologists [1]. These models excel at identifying subtle patterns and features that may pose challenges for the human eye, resulting in more accurate and reliable classifications [18,21]. Machine learning models mitigate the variability inherent in manual cell classification by delivering consistent results. Their efficiency accelerates the cell analysis process, which is particularly crucial in clinical settings where prompt diagnosis is imperative [17].

Additionally, these models can be trained on diverse datasets and adapt to various tissue types and staining methods, showcasing their versatility across a broad spectrum of applications [22]. The CNN and SVM models represent a neural network architecture characterized by the local coupling of common weights between layers, showing a paradigm shift in processing and classification in medical and dental histopathological and radiological images [16]. Machine learning and Artificial intelligence are used for cancer prediction, prognostication, and quality of life estimation [22-25].

Jansen et al. [26] have reported that digital slides have enabled the incorporation of pathology into patient management systems. It can also be visualized with three-dimensional 3D reconstruction to visualize tumor growth and could predict its behavior. Computer-aided diagnosis can help the pathologist in decision-making. Deep learning could be used to automate the classification of diseases and tumor grades, as inter-observer variability is usually high in such instances [26]. Deep learning has been successfully tested in histopathological cancer evaluation, including Ki-67 labeled mitotic activity, micro-metastasis in sentinel lymph nodes, and Gleason scoring of prostate biopsies [27]. Ana et al. [28] have reported Pathologist Computer-Aided Diagnostic Scoring of Tumor Cell Fraction: A Swiss National Study. They demonstrated improved scoring accuracy, interpathologist agreement, and scoring confidence. Interestingly, pathologists also expressed more willingness to use such a tool at the end of the survey, highlighting the importance of training/education to increase the adoption of AI systems.

Cell segmentation, which assesses the nucleus, cytoplasm, and structure of cells, varies with each AI model, and the outcome is based on the training data fed to the system [27]. We have started by assessing nuclear features using the SVM and CNN models. We are also pursuing cytoplasmic changes and measuring cellular area not only in cytology but also in histopathological sections. We are also collecting training data to compare different cytopathological and histopathological stains. Cellular and tissue changes with smoked tobacco and smokeless tobacco are also being explored. Cytological changes in various grades of dysplasia and different grades of squamous cell carcinomas are also planned in our research agenda. Outcome evaluation of AI models compared to trained oral pathologists for suspicious cellular changes will enable standardization of observed results. We are hoping that this initiative will ultimately end in a prompt diagnosis of oral cancer.

Although the current study suggests a potent use of artificial intelligence in medical and dental diagnosis, a few limitations must be addressed before concluding the results. The sample size, though representative, may not encompass the full spectrum of oral cancer variations. Moreover, variations in image quality, staining techniques, and patient demographics could influence feature extraction accuracy. Grad-CAM (Gradient-weighted Class Activation Mapping) and attention heatmap indicate the next step in research. Visualizing model classifications using these techniques will be valuable for the detailed analysis of nuclear parameters in cytopathological images.

## Conclusions

Both learning models were able to effectively differentiate between epithelial and dysplastic nuclear changes. Automated scrutiny of nuclear parameters in oral exfoliative cytology through machine learning presents significant potential for enhancing both the precision and expeditiousness of oral cancer diagnosis. The discerned diagnostic accuracy exhibited by the SVM and CNN models suggests prospective improvements in early detection rates. Nevertheless, the study underscores the latent utility of an automated analysis tool for oral cancer diagnosis reliant on nuclear parameters in exfoliative cytology images. Subsequent research might contemplate dataset expansion, thorough exploration of feature correlations, and comprehensive model validation across more extensive and diverse patient cohorts.
